# Cumulative effects of piscivorous colonial waterbirds on juvenile salmonids: A multi predator-prey species evaluation

**DOI:** 10.1371/journal.pone.0272875

**Published:** 2022-08-10

**Authors:** Allen F. Evans, Quinn Payton, Nathan J. Hostetter, Ken Collis, Bradley M. Cramer, Daniel D. Roby

**Affiliations:** 1 Real Time Research, Inc., Bend, Oregon, United States of America; 2 U.S. Geological Survey, North Carolina Cooperative Fish and Wildlife Research Unit, Department of Applied Ecology, North Carolina State University, Raleigh, North Carolina, United States of America; 3 Department of Fisheries, Wildlife, and Conservation Sciences, Oregon State University, Corvallis, Oregon, United States of America; Texas A&M University, UNITED STATES

## Abstract

We investigated the cumulative effects of predation by piscivorous colonial waterbirds on the survival of multiple salmonid (*Oncorhynchus* spp.) populations listed under the U.S. Endangered Species Act (ESA) and determined what proportion of all sources of fish mortality (1 –survival) were due to birds in the Columbia River basin, USA. Anadromous juvenile salmonids (smolts) were exposed to predation by Caspian terns (*Hydroprogne caspia*), double-crested cormorants (*Nannopterum auritum*), California gulls (*Larus californicus*), and ring-billed gulls (*L*. *delawarensis*), birds known to consume both live and dead fish. Avian consumption and survival probabilities (proportion of available fish consumed or alive) were estimated for steelhead trout (*O*. *mykiss*), yearling Chinook salmon (*O*. *tshawytscha*), sub-yearling Chinook salmon, and sockeye salmon (*O*. *nerka*) during out-migration from the lower Snake River to the Pacific Ocean during an 11-year study period (2008–2018). Results indicated that probabilities of avian consumption varied greatly across salmonid populations, bird species, colony location, river reach, and year. Cumulative consumption probabilities (consumption by birds from all colonies combined) were consistently the highest for steelhead, with annual estimates ranging from 0.22 (95% credible interval = 0.20–0.26) to 0.51 (0.43–0.60) of available smolts. The cumulative effects of avian consumption were significantly lower for yearling and sub-yearling Chinook salmon, with consumption probabilities ranging annually from 0.04 (0.02–0.07) to 0.10 (0.07–0.15) and from 0.06 (0.3–0.09) to 0.15 (0.10–0.23), respectively. Avian consumption probabilities for sockeye salmon smolts was generally higher than for Chinook salmon smolts, but lower than for steelhead smolts, ranging annually from 0.08 (0.03–0.22) to 0.25 (0.14–0.44). Although annual consumption probabilities for birds from certain colonies were more than 0.20 of available smolts, probabilities from other colonies were less than 0.01 of available smolts, indicating that not all colonies of birds posed a substantial risk to smolt mortality. Consumption probabilities were lowest for small colonies and for colonies located a considerable distance from the Snake and Columbia rivers. Total mortality attributed to avian consumption was relatively small for Chinook salmon (less than 10%) but was the single greatest source of mortality for steelhead (greater than 50%) in all years evaluated. Results suggest that the potential benefits to salmonid populations of managing birds to reduce smolt mortality would vary widely depending on the salmonid population, the species of bird, and the size and location of the breeding colony.

## Introduction

Predation shapes communities and affects persistence of prey populations [1]. In the Columbia River basin (CRB), USA, several anadromous Pacific salmonid (*Oncorhynchus* spp.) species and populations (Evolutionarily Significant Units [ESUs] or Distinct Population Segments [DPSs]; hereafter ESU) are listed under the U.S. Endangered Species Act (ESA). Each ESU reflects a unique lineage, but many life-history traits are similar across populations [2]. For example, all Pacific salmonids originating in the Snake River out-migrate as juveniles through the lower Snake and Columbia rivers during the spring and early summer. These shared life-history traits may lead to similar drivers of population dynamics (i.e., factors influencing survival, reproduction); however, species-specific traits (e.g., fish size at time of out-migration) may also influence the effects of specific stressors [3].

Previous research has identified predation by Caspian terns (*Hydroprogne caspia*, hereafter “terns”) and double-crested cormorants (*Nannopterum auritum*, hereafter “cormorants”) and consumption by California gulls (*Larus californicus*) and ring-billed gulls (*L*. *delawarensis*, collectively hereafter “gulls”) as a substantial mortality factor for some salmonid species and populations in the CRB in some years [4–7]. Previous studies were largely focused on quantifying the effects of individual breeding colonies [4–6, 8, 9]; however, most salmonid populations, like those originating from the Snake River, must migrate through the foraging ranges of breeding birds from multiple colonies during seaward migration [6, 10]. In addition to predation from piscivorous colonial waterbirds, salmonids are subjected to numerous other non-avian sources of mortality during out-migration. For example, mortality associated with hydroelectric dam passage, predation by piscivorous fish, disease, and other factors have been documented in the CRB [11–13]. Determining what proportion of total mortality (1 –survival) is attributable to avian predation may be critical for prioritizing recovery actions for ESA-listed salmonid populations in the CRB.

Capture-recapture-recovery studies are often used to identify and quantify specific sources of mortality in anadromous juvenile salmonids or smolts [6, 9, 12, 14]. These studies rely on marking (tagging) fish and then using subsequent recapture and recovery events to estimate survival and cause-specific mortality (e.g., harvest, dam passage, predation). Using this analytical framework, a previous study [6] provided evidence that the cumulative effects of predation by multiple bird species from several different colonies on ESA-listed Upper Columbia River steelhead trout *(O*. *mykiss)* were substantial, with predation probabilities more than 0.25 (i.e., 25%) of available smolts in some years. Comparisons of total steelhead mortality to mortality due to predation by colonial waterbirds indicated that avian predation accounted for 42–70% of all mortality sources [7]. Predation effects have been documented across larger river reaches or segments where piscivorous waterbirds from up to 12 different breeding colonies foraged on steelhead smolts [6, 7]. Several other previously published studies indicate that steelhead smolts are particularly susceptible to colonial waterbird predation, with predation impacts on steelhead often significantly higher than those observed on salmon (e.g., Chinook salmon [*O*. *tshawytscha*] and sockeye salmon [*O*. *nerka*]) smolts when controlling for bird colony and year [4, 6, 8]. Given the greater susceptibility of steelhead smolts to colonial waterbird predation observed in these studies, we predict that the cumulative effects of avian predation on salmon smolts are lower than those on steelhead smolts for Snake River populations. Research to quantify cumulative predation probabilities for multiple Snake River salmonid populations, however, are generally lacking in the published literature but may be paramount to designing and evaluating predator management actions to benefit salmonid species and populations [7, 15].

We investigated the cumulative effects of avian predation across multiple ESA-listed salmonid species and populations that migrate through the foraging range of piscivorous waterbirds belonging to several species and nesting at different colonies to quantify what proportion of total smolt mortality was due to avian predation. We conducted analyses on four different populations of juvenile salmonids tagged with passive integrated transponder (PIT) tags and originating from the Snake River basin: (1) steelhead trout, (2) yearling Chinook salmon, (3) sub-yearling Chinook salmon, and (4) sockeye salmon. Survival and avian consumption probabilities were evaluated across multiple river reaches (spatial scales) during an 11-year study period (2008–2018). Results provide a novel, comprehensive evaluation of predation and survival across multiple ESA-listed salmonid populations and spatial-scales.

## Materials and methods

### Study area

**Fig 1:**
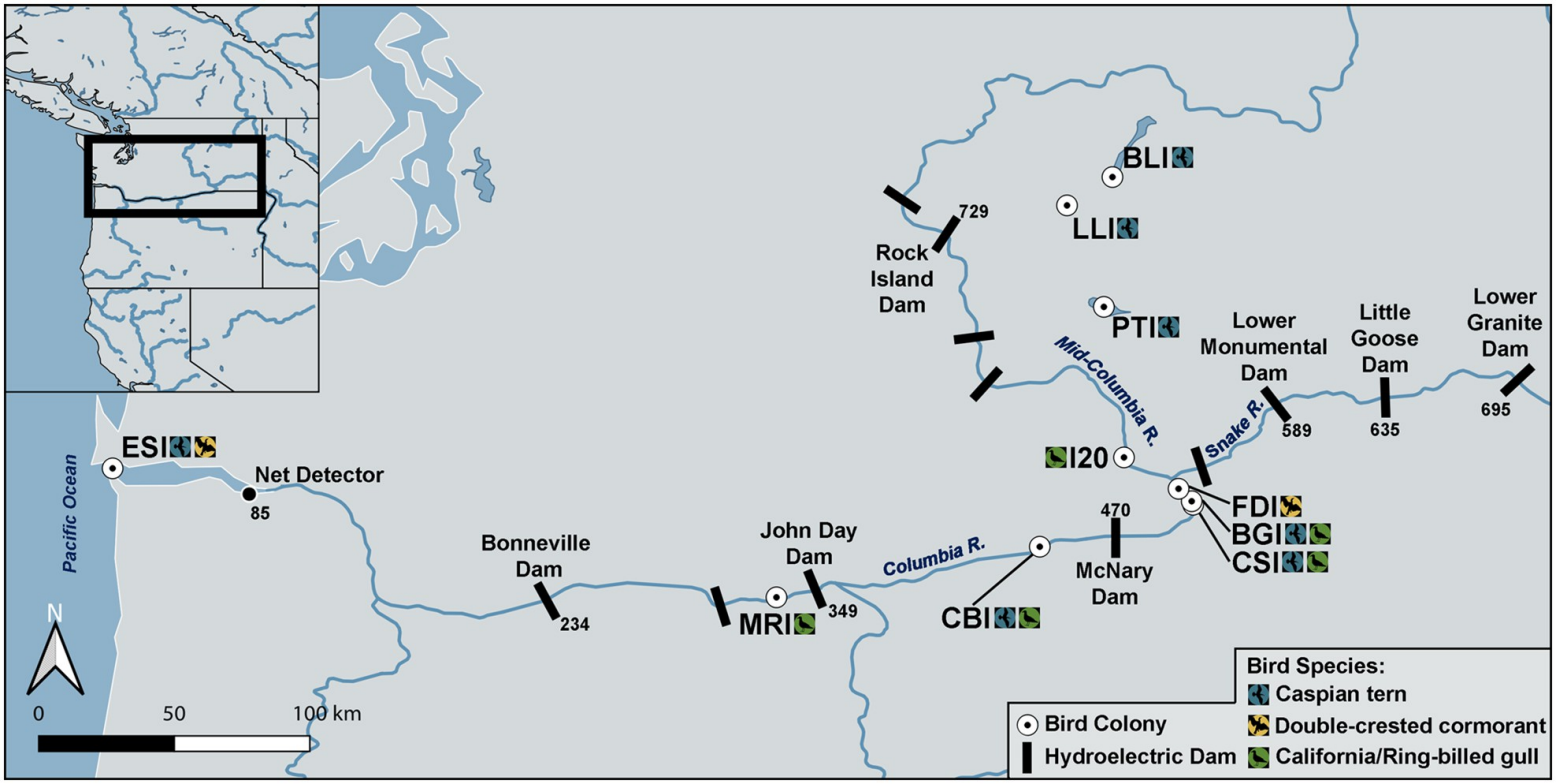
Map of study area. Capture-recapture-recovery locations of tagged juvenile salmonids initially detected passing Lower Granite Dam on the Snake River. Downstream recapture locations include Little Goose Dam, Lower Monumental Dam, McNary Dam, John Day Dam, Bonneville Dam, and a towed net detection system in the upper Columbia River estuary (Net Detector). Recovery locations include bird colonies on Banks Lake Island (BLI), Potholes Reservoir (PTI), Lenore Lake Island (LLI), Island 20 (I20), Foundation Island (FDI), Badger Island (BGI), Crescent Island (CSI), central Blalock Islands (CBI), Miller Rocks Island (MRI), and East Sand Island (ESI). Avian species include Caspian terns, double-crested cormorants, and California/ring-billed gulls. Numbers represent the distance in river km (Rkm) from the Pacific Ocean.

Our study focused on predation and survival probabilities for salmonid smolts out-migrating through the Snake River (SR) and Columbia River (CR) to the Pacific Ocean, a distance of 695 Rkm (Fig 1). The study area was divided into multiple river reaches or segments, including from (1) Lower Granite Dam to Little Goose Dam, a 60 Rkm section of SR, (2) Little Goose Dam to Lower Monumental Dam, a 46 Rkm section of SR, (3) Lower Monumental Dam to McNary Dam, a 119 Rkm section of the SR and CR, (4) McNary Dam to John Day Dam, a 121 Rkm section of the CR, (5) John Day Dam to Bonneville Dam, a 115 Rkm section of the CR, and (6) Bonneville Dam to the Pacific Ocean, a 234 Rkm section of the CR (Fig 1).

### Capture-recapture-recovery data

We integrated multiple data sources to estimate avian consumption and survival probabilities (proportion of available tagged fish) for smolts that were marked with PIT tags. Data sources included fish PIT-tagged at or interrogated passing (i.e., previously tagged and detected) Lower Granite Dam on the Snake River, detections of these fish at downstream recapture sites, and recoveries of tags from consumed fish on bird colonies (Fig 1). Juvenile salmonids were PIT-tagged, assigned a species (steelhead, Chinook salmon, sockeye salmon) and age-class (yearling or sub-yearling, for Chinook salmon only), and subsequently released to continue their out-migration as part of independent smolt behavior and survival studies in the region [16–18]. PIT-tagged smolts were detected at Lower Granite Dam from early-April to late-July each year, with the duration of the detection period dependent on population-specific run-timing. We evaluated four unique groups of PIT-tagged Snake River salmonids, referred to herein as populations: (1) steelhead trout, (2) yearling Chinook salmon, (3) sub-yearling Chinook salmon, and (4) sockeye salmon. Tagged populations included both hatchery and wild (natural origin) fish. Not all hatcheries are included in the ESA-defined ESUs, and in the case of Chinook salmon, yearlings may be a mixture of two distinct ESA-listed populations (spring- and fall-run; NOAA 2014); thus, populations in this study reflect the same species and age-classes but some unknown proportion of fish may not be part of the ESA-listed ESU or were a mixture of two ESA-listed ESUs.

Following their initial capture/recapture at Lower Granite Dam, tagged fish were passively detected passing downstream sites outfitted with in-stream PIT tag arrays located at hydroelectric dams or the net detector in the estuary (Fig 1). Only naturally or volitionally out-migrating smolts within each river reach were included, with all transported smolts excluded following, but not prior to, their removal from the river in fish barges or trucks (see *below* for additional details). Adults returning to the Columbia River following ocean residency were also passively detected at PIT tag arrays located in fishways at Bonneville Dam, the first dam encountered by SR adults following ocean residency. Release and recapture records were retrieved from the PIT Tag Information System, a regional mark, recapture, recovery database maintained by the Pacific States Marine Fisheries Commission [18].

Smolt PIT tags were also recovered on piscivorous waterbird colonies located throughout the study area (Fig 1). Recoveries from a total of 14 unique bird colonies were included in the study, colonies that were previously identified as posing a potential threat to smolt survival [6–7]. The methods of Evans et al. [6] were used to recover PIT tags from each bird colony. In brief, portable PIT tag antennas were used to detect tags *in situ* after birds dispersed from their breeding colonies at the end of the nesting season (August–October). The entire land area occupied by nesting birds was scanned for tags following each breeding season, with a minimum of two complete sweeps or passes of each colony site conducted each year. The land area occupied by birds during each breeding season was determined based on aerial photography surveys and/or ground surveys of the colony carried out during the peak of the breeding season from late-May to early-June. Counts of the number of breeding adults on each colony were generated from aerial and ground surveys as part of separate studies and are provided in Evans et al. [7].

Not all smolt PIT tags ingested by birds are deposited on the bird’s nesting colony (i.e., deposition probabilities for consumed tags were less than 1.0) and not all tags deposited on the colony are subsequently detected by researchers after the nesting season (i.e., detection probabilities for deposited fish tags were less than 1.0; [19]). To incorporate predator- and colony-specific PIT tag deposition and detection probabilities, collectively referred to as recovery probabilities (deposition × detection), we incorporated summarized results from previously published studies which estimated the likelihood of tags being recovered given these processes [7, 19]. In brief, data on deposition probabilities were based on recoveries of PIT tags from smolts that were intentionally fed to nesting terns, cormorants, and gulls at multiple colonies and years, and the recoveries of these known ingested tags were used to estimate deposition probabilities [19]. Results indicated that deposition probabilities varied significantly by predator species (terns, cormorants, gulls) but that there was no significant difference in deposition probabilities within years, between years, or between colonies of the same species [19]. Unlike deposition probabilities, results of detection probability studies indicated that detection varied within and between years, necessitating empirically derived estimates of detection probability at each colony, in each year [7, 15]. To estimate detection probabilities, as part of independent studies, PIT tags were intentionally sown on each bird colony by researchers prior to, during (when possible), and following the nesting season at each of the colonies included in this study, in each year [7]. Recoveries of sown tags during scanning efforts after the nesting season were then used to model the probability of detecting a tag that was deposited on the colony during the breeding season (see *below* for additional details).

Not all colony sites had nesting birds in all study years, nor were all sites scanned for smolt PIT tags in all years. Specifically, gull colonies on Island 20 in McNary Reservoir and the Blalock Islands in John Day Reservoir were not scanned for PIT tags during 2008–2012, so estimates of consumption by gulls from those colonies in those years were not generated. The cormorant colony on Foundation Island in McNary Reservoir was not scanned for smolt PIT tags during 2013 or 2015–2018, so estimation of consumption by cormorants were not generated in those years. Cormorants also temporarily dispersed (abandoned) their nesting colony site on East Sand Island in the Columbia River estuary during the peak of the smolt out-migration period of April to June each year during 2016–2018 [7]. As such, although the cormorant colony was scanned for PIT tags in all years following each breeding season, PIT-tagged smolts that were consumed by cormorants during dispersal events were presumably deposited off-colony, resulting in minimum estimates of annual consumption probabilities in those years. Unlike gull and cormorant colonies, all active tern colonies were scanned for smolt PIT tags in all study years.

Finally, predation by terns and cormorants is generally considered to occur on live fish [20, 21]. Gulls, however, are known to consume live fish and to scavenge dead fish [22, 23], so it is unknown what proportion of consumed smolts by gulls were dead or moribund when consumed. As such, we use the term “consumption probabilities” when referring to the effects of piscivorous colonial waterbirds on the survival of juvenile salmonids in this study. Consumption probabilities are statistically analogous to predation probabilities reported in other studies [7, 15, 19].

### Consumption and survival estimation

The joint mortality and survival (JMS) estimation methods of Payton et al. [15] were used to independently estimate colony-specific, reach-specific, and cumulative consumption and survival probabilities for each salmonid species and population (hereafter simply “population”) evaluated. This hierarchal state-space Bayesian model incorporated both live and dead detections of PIT-tagged fish in space and time to simultaneously estimate consumption and survival through nine sequential river reaches (or segments), defined by passive recapture opportunities in which smolts were assumed to only travel downstream. In brief, the model used two vectors, ***y*** and **r**, to describe each fish’s recapture and recovery history throughout each of the 9 downstream river reaches (delineated by 8 potential recapture sites) and each of the 14 bird colony recovery sites under consideration. Each vector **y** was a 9-length vector, where *y*_*j*_ was an indicator variable of a fish’s recapture at recapture opportunity *j* for *j* ∈ {1,2, …, *J* − 1 = 8} and *y*_9_ = 0 as there was no recapture site downstream of the net detector in the Columbia River estuary (Fig 1). Recoveries were indicated by **r**, a 15-length vector with a single element equal to one and the rest of the elements are zero, where *r*_*d*_ = 1 indicated recovery on colony *d* for *d* ∈ {1,2, …, *D* − 1 = 14}, and *r*_15_ = 1 indicated a fish was not recovered. Parameters used in the model included:

**Θ**, a 15x5 matrix where *Θ*_*j*,*d*_ represented the probability (from release) that a fish survived to recapture opportunity *j* and then subsequently succumbed to depredation by colony *d* for *d* ∈ {1,2, …,14} or some other cause of mortality for *d* = 15, prior to arrival at recapture opportunity *j* + 1. Implicit from this parameterization is that survival from release through segment *k* is equal to 1 − Σ_*j*≤*k*_ Σ_*d*_ Θ_*j*,*d*_.
**p**, a 9-length vector where *p*_*j*_ represented the probability that a fish alive at recapture opportunity *j* was successfully recaptured. We define *p*_9_ = 0, as there is no recapture opportunity downstream of the Net Detector.
**γ**, a 15-length vector where *γ*_*d*_ represented the probability of recovering a fish which died due to depredation by colony *d* for *d* ∈ {1,2,…,14}, and γ_15_ = 0 represented the lack of recovery opportunity for fish which died from all other unspecified causes.

The model employed can be expressed by incorporating these parameters into recursive functions, *χ*_*j*,*d*_, defined to represent the probability a fish entering segment *j* is not subsequently recaptured and is recovered on colony *d* (i.e., *r*_*d*_ = 1), such that

$$\chi_{j,d}=\theta_{j,d}*\gamma_{d}+\left(1-p_{j+1}\right)*\chi_{j+1,d}\;for\;d\in 1,\ldots ,14,$$

or not recovered at all (i.e., *r*_15_ = 1), such that

$$\chi_{j,D}=\sum_{d}\theta_{j,d}*\left(1-\gamma_{d}\right)+\left(1-p_{j+1}\right)*\chi_{j+1,D}.$$

Then, if we define *m* to be the final recapture opportunity at which the fish was seen, with *m* = 0 representing a fish never reseen following release, the portion of the aggregate likelihood associated with each fish’s recapture/recovery history can be expressed as

$$L=\prod_{j\leq m}\left(p_{j}^{y_{j}}*{\left(1-p_{j}\right)}^{\left(1-y_{j}\right)}\right)*\prod_{d}\chi_{m+1,d}{}^{r_{d}},$$

where the former product describes a fish’s recapture history prior to its final recapture and the latter product describes the fish’s subsequent recovery or lack thereof following its final recapture.

Each year, a small subset of tagged smolts were collected and removed from the river in fish barges or trucks at one of the first three capture/recapture sites on the lower Snake River: Lower Granite Dam, Little Goose Dam, or Lower Monumental Dam. Once collected for transportation, these fish were no longer available in-river and, as such, the capture-recapture-recovery history for these fish was truncated following their removal at each dam. The likelihood associated with the truncated capture-recapture history of each of these fish can be expressed as:

$$L=\prod_{j<m}\left(p_{j}^{y_{j}}*{\left(1-p_{j}\right)}^{\left(1-y_{j}\right)}\right)*\left(1-\sum_{d}\sum_{j<m}\theta_{j,d}\right)$$

To measure inner-annual temporal variation in probabilities, fish were partitioned into weekly release groups with the assumption that fish released within the same week experienced similar rates of mortality/survival, recapture, and recovery, smolts from each salmonid population were grouped into weekly release cohorts. While all rates were assumed to be independent among years, weekly cohorts closer in time were assumed to be more alike than those further apart. Previous research has suggested temporal autocorrelation to be inherent to all rates of mortality/survival [19], recapture [24], and recovery [6, 8]. The serial correlation in probabilities were assumed and accounted for as described by Payton et al. [15]. The prior distribution for the initial week’s detection probability in each year was defined to be uniform(0,1). Analogously, the prior distribution assigned for the life paths simplexes in the initial week of each year was assumed to be dirichlet(**1**), where **1** was an appropriately sized vector of ones. Weakly-informative priors of half-normal(0,5) were implemented for the variance parameters describing inter-weekly variation.

The JMS model partitions the effects from colony *d* among the river reaches from which that colony is known to forage. The columns of, **Θ**, represent the cumulative probability from release that a fish succumbs to depredation due to colony *d* for *d* ∈ 1,…,14, with the 15^th^ column representing cumulative probability that a fish succumbs to another unspecified cause of mortality. The vector [*θ*_1,*d*_, *θ*_2,*d*_, *θ*_*J*,*d*_]^*T*^ therefore represents a partitioning of the cumulative probability of death due to *d*. In this study, at most two river reaches are foraged by any given colony, colonies may not be active in every year, and we assume that in each year they are active they forage among the same river reaches (as was the case in Payton et al. [15]). The low recapture rates (inherent to juvenile bypass detection of PIT tagged smolts) inhibits precision in this partitioning. Previous research indicates that consumption impacts from individual colonies were spatially proportionate amongst river reaches across years [25, 26]. We therefore implemented an “informed partitioning” method to share information among years and increase precision in estimates of reach-specific predation probabilities. This approach represents a novel augmentation of the methods of Payton et al. [15].

We defined $\theta_{d}^{cumulative}=1^{T}\theta_{d}$, to be the cumulative probability that a fish succumbs to *d*, and let *J* -length vector ***ρ***_*d*_ define the proportion of $\theta_{d}^{cumulative}$ associated with each segment such that probabilities, so that

$${\left[\theta_{1,d},\theta_{2,d},\ldots ,\theta_{J,d}\right]}^{T}=\theta_{d}^{cumulative}\rho_{d}^{T}.$$

To “share” information among years, we assumed each year’s ***ρ***_*d*_ vector to be selected from a colony specific Dirichlet hyper-distribution,

$$\rho_{d}\sim \text{dirichlet}\left(\alpha_{d}\right)$$

where ***α***^*d*^ defines the average odds that birds from colony *d* forage within each segment across years. Weakly-informative priors for the ***α***_*d*_ vectors of Gamma (2, 4) were implemented (as suggested by [27]) for each non-zero element of ***α***_*d*_ to reflect an unbiased assumption of proportionality across the segment’s colony *d* was known to forage within.

The recovery parameters, *γ*_*d*_, represent the combined probability that a consumed tag was deposited on-colony, *d*, and the probability that the tag is subsequently detected (recovered) by researchers following the breeding season given deposition on a colony. The simulated posterior distributions of deposition probabilities and colony-specific detection probabilities which were derived, summarized, and presented in previous studies were employed here as informative prior distributions in the derivation of predation probability estimates. Informative prior distributions used in this study are provided as S1 File (see also Evans et al. [7] for recovery probabilities from each colony in each year).

Models were analyzed using the software STAN [28], accessed through R version 3.6.2 [29], and using the rstan package (version 2.19.3; [28]). To simulate random draws from the joint posterior distribution, we ran four Hamiltonian Monte Carlo (HMC) Markov Chain processes. Each chain contained 4,000 warm-up iterations followed by 4,000 posterior iterations thinned by a factor of 4. Chain convergence was visually evaluated and verified using the Gelman-Rubin statistic [30]; only chains with zero reported divergent transitions were accepted. Posterior predictive checks compared simulated and observed annual aggregate raw recapture and recovery numbers to ensure model estimates reflected the observed data. Reported estimates represent simulated posterior medians along with 95% highest (posterior) density intervals (95% Credible Interval [CRI]) calculated using the HDInterval package (version 0.2.0; [31]).

No animals (i.e., birds or fish) were handled as part of this retrospective analysis, with data made available to us in a regional capture, recapture, recovery database [18] or as part of previously published studies [7, 19].

## Results

### Capture-recapture-recovery

Sample sizes of PIT-tagged smolts used in analyses varied considerably by salmonid population and year (Table 1). In total, 713,877 steelhead, 1,044,755 yearling Chinook salmon, 150,351 sub-yearling Chinook salmon, and 31,220 sockeye salmon were detected passing Lower Granite Dam during 2008–2018. Annual sample sizes ranged from 298 tagged sockeye smolts in 2008 to 167,925 tagged yearling Chinook salmon smolts in 2009 (Table 1). The number of smolts subsequently detected alive (recaptured) at downstream PIT tag arrays or recovered dead on bird colonies also varied by salmonid population and year (Table 1). In total, 57,433 tagged steelhead smolts, 27,444 tagged yearling Chinook salmon smolts, 2,525 tagged sub-yearling Chinook salmon smolts, and 723 tagged sockeye salmon smolts were recovered on bird colonies during 2008–2018 (Table 1). Annual numbers of recovered tags ranged from 5 sockeye salmon tags in 2017 to 12,467 steelhead tags in 2009 (Table 1). A relatively small number and proportion of PIT-tagged smolts returned to Bonneville Dam as adults, with smolt-to-adult return (SAR) rates ranging from 0.2–3.0%, depending on the salmonid population and year (Table 1).

**Table 1:** Numbers of passive integrated transponder (PIT)-tagged smolts used in this study. Numbers are by species/population and year that were detected/released (Rel) passing Lower Granite Dam; that were subsequently detected/recaptured downstream alive (Live); that were recovered on a bird colony (Dead); or were detected returning as an adult to Bonneville Dam (SAR). NA denotes that complete adult returns were not available. The same fish could be detected multiple times at downstream detection/recapture sites (Live), but only one detection was possible at a recovery site (Dead).

Year	Steelhead (Rel-Live-Dead-SAR)	Yearling Chinook (Rel-Live-Dead-SAR)	Sub-yearling Chinook (Rel-Live-Dead- SAR)	Sockeye (Rel-Live-Dead-SAR)
2008	53155–50934–6822–1481	156901–131520–4336–2471	22032–12284–469–342	298–265–17–2
2009	89445–100983–12467–1404	167925–160002–8246–1706	21347–12705–444–76	3009–2407–111–45
2010	45348–29304–5318–615	136085–79256–4501–803	21075–14605–314–309	1656–806–58–9
2011	80903–86392–6427–606	134728–136283–3208–714	36537–19296–596–604	8250–4543–169–26
2012	78040–74315–5085–1336	96343–93182–1439–1831	25286–12486–429–318	4322–3326–113–16
2013	42953–25868–2859–514	42410–30711–649–543	2529–1132–47–30	5521–2336–92–120
2014	64725–47813–5285–799	75662–69988–1896–483	3009–1859–68–15	1656–837–69–14
2015	40715–16950–3198–98	27792–12472–1046–118	3718–980–39–NA	1019–331–16–2
2016	79262–66351–3783–NA	102858–97031–1348–NA	5203–1694–32–NA	1721–823–14–1
2017	75019–57734–3744–NA	56829–42363–445–NA	5251–2386–26–NA	451–170–5–NA
2018	64312–52118–2445–NA	47222–34233–330–NA	4364–2544–61–NA	3317–1440–59–NA

### Consumption and total mortality

Estimates of total mortality (1 –survival) between Lower Granite Dam and Lower Monumental Dam ranged annually from 0.09 (95% credible interval = 0.08–0.10) to 0.20 (0.19–0.22) in steelhead smolts, from 0.06 (0.04–0.10) to 0.24 (0.22–0.26) in yearling Chinook smolts, from 0.11 (0.03–0.20) to 0.33 (0.31–0.34) in sub-yearling Chinook smolts, and from 0.03 (0.01–0.11) to 0.43 (0.40–0.47) in sockeye smolts. Result indicated that the majority of smolts survived passage from Lower Granite Dam to Lower Monumental Dam. Avian predation effects were first observed in the river reach located downstream of Lower Monumental Dam, indicating there was no measurable consumption of smolts between Lower Granite Dam and Little Goose Dam and Little Goose Dam and Lower Monumental Dam associated with the bird colonies included in the study. Estimates of avian consumption and total mortality downstream of Lower Monumental Dam varied considerably by bird species, colony location, salmonid population, river reach, and year and are provided below.

#### Steelhead trout

**Fig 2:**
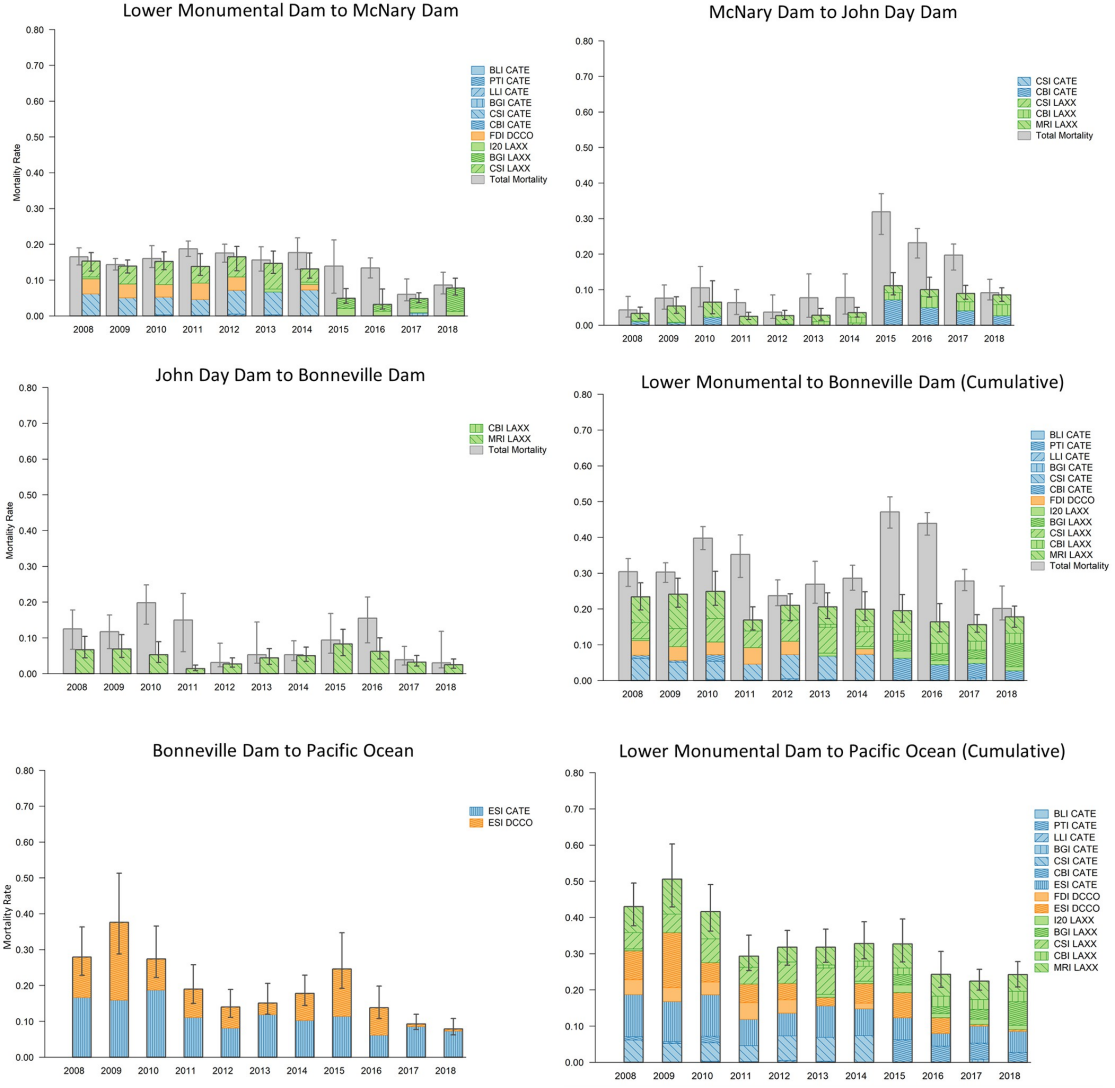
Avian consumption and total mortality of snake river steelhead. Estimated annual reach-specific and cumulative avian consumption probabilities (colored bars) and total mortality (grey bars) of Snake River steelhead trout smolts during passage from Lower Monumental Dam on the Snake River to the Pacific Ocean. Piscivorous waterbird colony locations include Banks Lake Island (BLI), Potholes Reservoir (PTI), Lenore Lake Island (LLI), Island 20 (I20), Foundation Island (FDI), Badger Island (BGI), Crescent Island (CSI), central Blalock Islands (CBI), Miller Rocks Island (MRI), and East Sand Island (ESI). Avian species include Caspian terns (CATE), double-crested cormorants (DCCO), and California and ring-billed gulls (LAXX). Error bars represent 95% credible intervals for total mortality and avian consumption.

Cumulative consumption probabilities (consumption by birds from all colonies combined) on steelhead smolts during passage from Lower Monumental Dam to the Pacific Ocean were substantial and were the highest among the four salmonid populations evaluated, ranging annually from 0.22 (0.20–0.26) to 0.51 (0.43–0.60; Fig 2). The evaluation of smolt mortality due to avian consumption in the context of total smolt mortality from Lower Monumental Dam to Bonneville Dam indicated that avian consumption accounted for 37% (95% credible interval = 33–46%) to 89% (80–100%) of all steelhead smolt mortality, with avian consumption accounting for > 50% of all smolt mortality in 9 of 11 years evaluated (Fig 2). Consumption probabilities were often the highest for tern colonies, with terns depredating 0.07 (0.05–0.09) to 0.17 (0.13–0.23) of all steelhead smolts annually (Fig 2). The magnitude of consumption by cormorants was often lower than that of terns, ranging annually from 0.06 (0.04–0.07) to 0.17 (0.11–0.26; Fig 2). The magnitude of steelhead consumption probabilities by gulls, which were limited to those nesting at colonies upstream of Bonneville Dam and foraging exclusively above Bonneville Dam, were also substantial, but often less than that of terns and cormorants nesting at nearby colonies; annual consumption probabilities for gulls ranged from 0.07 (0.05–0.10) to 0.13 (0.10–0.17) (Fig 2).

Of the individual bird colonies capable of consuming smolts during passage from Lower Monumental Dam to McNary Dam, consumption probabilities for steelhead were greatest for terns nesting on Crescent Island in McNary Reservoir during 2008–2014, with estimated of 0.04 (0.03–0.06) to 0.07 (0.05–0.11) of available steelhead smolts depredated annually (Fig 2). Consumption probabilities for terns from the other three tern colonies that could potentially forage in this reach, those at Potholes Reservoir, Lenore Lake Island, and Banks Lake Island, were less than 0.01 (Fig 2). Terns from these other three colonies were nesting between 73 and 129 km from the nearest section of the Snake River downstream of Lower Monumental Dam (Fig 1). Consumption probabilities for cormorants nesting on Foundation Island in McNary Reservoir were like those of terns nesting on Crescent Island, also in McNary Reservoir, ranging annually from 0.03 (0.02–0.05) to 0.05 (0.03–0.07) during 2008–2012 and 2014 (Fig 2), the six years when the cormorant colony was scanned for smolt PIT-tags (see Methods). Of the individual gull colonies evaluated, consumption probabilities were highest for gulls nesting on Crescent Island during 2008–2014, prior to colony management to eliminate the colony in 2015 (see also [7]), ranging from 0.03 (0.02–0.05) to 0.07 (0.05–0.10; Fig 2). Consumption probabilities for gulls nesting on Island 20, located on the middle Columbia River 20 km upstream of the confluence of the Snake River, were the lowest of the gull colonies evaluated at less than 0.02 of available smolts (Fig 2).

Consumption probabilities for steelhead smolts during passage from McNary Dam to John Day Dam on the Columbia River were greatest for the tern colony on the Blalock Islands in John Day Reservoir during 2015–2018, with probabilities as high as 0.07 (0.05–0.10), followed by the consumption probabilities for the gull colony on the Blalock Islands at 0.03 (0.02–0.04; Fig 2). Nearly all consumption of steelhead smolts during passage from John Day Dam to Bonneville Dam was due to the gulls nesting at the colony on Miller Rocks Island in The Dalles Reservoir, with estimates ranging annually from 0.02 (0.01–0.02) to 0.08 (0.05–0.12), amongst the highest consumption probabilities of the four gull colonies evaluated in the study (Fig 2). During smolt passage from Bonneville Dam to Pacific Ocean, consumption probabilities for terns nesting on East Sand Island in the Columbia River estuary were consistently greater than 0.10, and as high as 0.19 (0.15–0.27), amongst the highest reach-specific estimates for any individual colony. Consumption probabilities for cormorants nesting on East Sand Island were also substantial in some, but not all years, ranging annually from 0.03 (0.02–0.05) to 0.21 (0.14–0.33) during 2008–2015, years when uninterrupted cormorant nesting behavior was observed on East Sand Island (see *Methods*).

#### Yearling Chinook salmon

**Fig 3:**
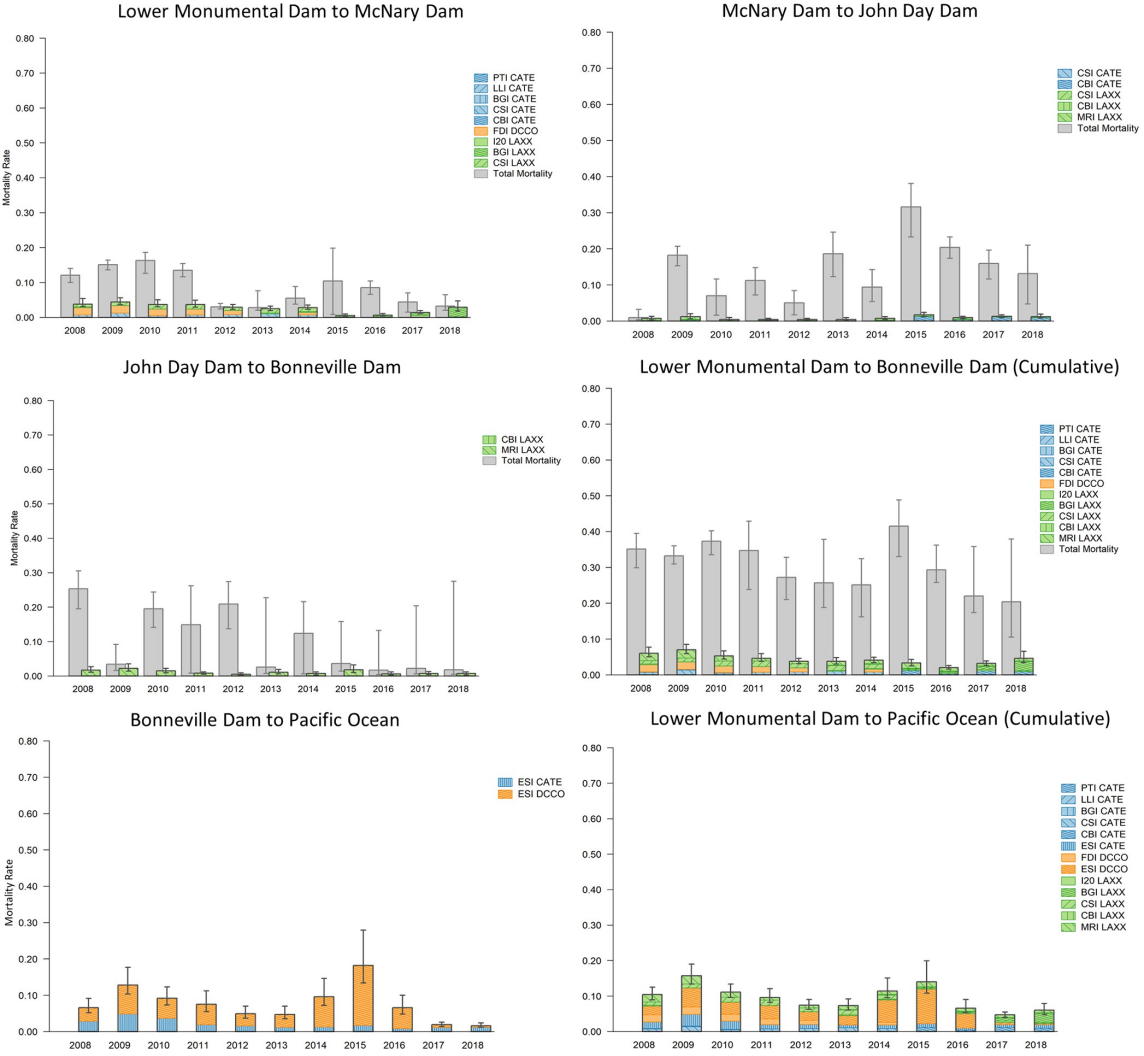
Avian consumption and total mortality of snake river yearling Chinook salmon. Estimated annual colony-specific, reach-specific, and cumulative avian consumption probabilities (colored bars) compared to total mortality (grey bars) of Snake River yearling Chinook salmon smolts during passage from Lower Monumental Dam on the Snake River to the Pacific Ocean. Piscivorous waterbird colony locations include Banks Lake Island (BLI), Potholes Reservoir (PTI), Lenore Lake Island (LLI), Island 20 (I20), Foundation Island (FDI), Badger Island (BGI), Crescent Island (CSI), central Blalock Islands (CBI), Miller Rocks Island (MRI), and East Sand Island (ESI). Avian species include Caspian terns (CATE), double-crested cormorants (DCCO), and California and ring-billed gulls (LAXX). Error bars represent 95% credible intervals for total mortality and avian consumption.

Cumulative consumption probabilities for yearling Chinook salmon smolts during out-migration from Lower Monumental Dam to the Pacific Ocean were significantly lower than those for steelhead smolts, ranging annually from 0.05 (0.04–0.06) to 0.16 (0.13–0.19; Fig 3). Comparisons of total mortality for yearling Chinook salmon smolts to mortality due to avian consumption indicated avian consumption accounted for 7% (4–19%) to 23% (9–37%) of all mortality during smolt passage from Lower Monumental Dam to Bonneville Dam (Fig 3). Of the river reaches evaluated, avian consumption accounted for the greatest proportion of total mortality during smolt passage from Lower Monumental Dam to McNary Dam, with avian consumption accounting for 7% (4–17%) to upwards of 97% (58–100%) of all smolt mortality annually (Fig 3). Avian consumption was consistently a smaller component of total smolt mortality from McNary Dam to John Day Dam and from John Day Dam to Bonneville Dam, with avian consumption often accounting for less than 20% of total mortality in these two river reaches in most years (Fig 3). The magnitude of avian consumption on yearling Chinook salmon smolts was greatest in the Columbia River estuary, with estimated annual probabilities ranging from 0.05 (0.04–0.07) to 0.18 (0.13–0.28; Fig 3); however, the magnitude of avian consumption could not be compared to total smolt mortality in this river reach because of the lack of survival estimates downstream of Bonneville Dam.

Of the individual colonies evaluated, consumption probabilities on yearling Chinook salmon smolts during passage from Lower Monumental Dam to McNary Dam were greatest for cormorants nesting on Foundation Island in McNary Reservoir (Fig 3). Consumption probabilities for Foundation Island cormorants and for other bird colonies in this reach, however, were less than 0.03 of available smolts annually (Fig 3). Similarly, consumption probabilities for yearling Chinook salmon smolts during passage from McNary Dam to John Day Dam were also low at less than 0.02 of available smolts, per colony, per year (Fig 3). Analogous to consumption of steelhead smolts, nearly all avian consumption of yearling Chinook salmon smolts during out-migration from John Day Dam to Bonneville Dam was due to gulls nesting on Miller Rocks; however, unlike consumption probabilities for steelhead smolts, probabilities for yearling Chinook salmon smolts were low at less 0.03 of available smolts annually (Fig 3). Despite low consumption probabilities observed for birds at colonies upstream of Bonneville Dam, avian consumption probabilities for yearling Chinook salmon smolts that reached the estuary were substantial and significantly higher, particularly consumption by cormorants nesting at the colony on East Sand Island, where consumption probabilities were consistently higher than 0.04, and as high as 0.17 (0.12–0.26), during 2008–2015 (Fig 3). Consumption probabilities for yearling Chinook salmon smolts by terns nesting on East Sand Island were consistently lower than those of cormorants nesting on East Sand Island but often higher than those of terns, cormorants, and gulls nesting at colonies upstream of Bonneville Dam, ranging annually from 0.01 (0.01–0.02) to 0.05 (0.04–0.07) during 2008–2018 (Fig 3).

#### Sub-yearling Chinook salmon

**Fig 4:**
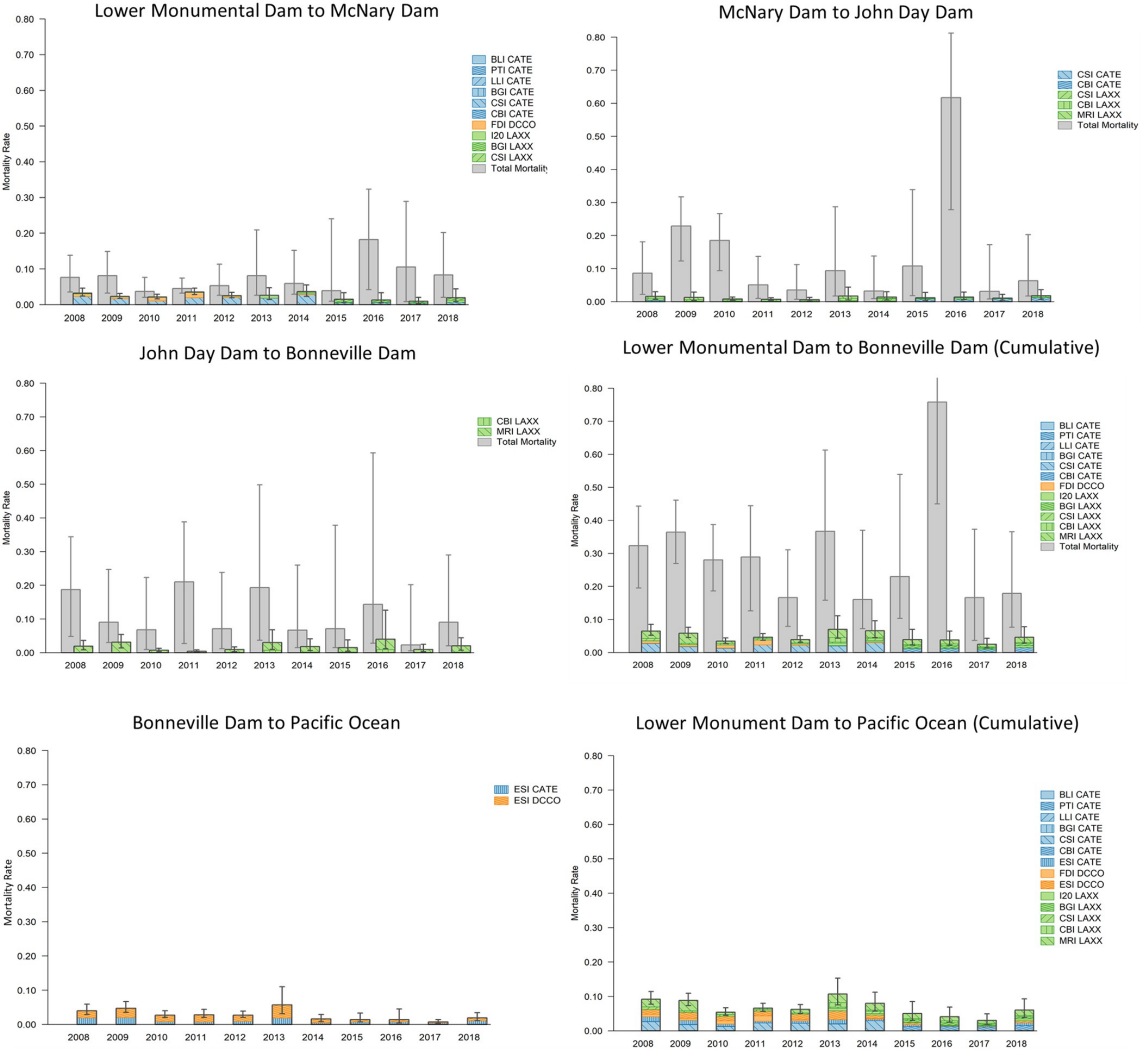
Avian consumption and total mortality of snake river sub-yearling Chinook salmon. Estimated annual colony-specific, reach-specific, and cumulative avian consumption probabilities (colored bars) and total mortality (grey bars) of Snake River sub-yearling Chinook salmon smolts during passage from Lower Monumental Dam on the Snake River to the Pacific Ocean. Piscivorous waterbird colony locations include Banks Lake Island (BLI), Potholes Reservoir (PTI), Lenore Lake Island (LLI), Island 20 (I20), Foundation Island (FDI), Badger Island (BGI), Crescent Island (CSI), central Blalock Islands (CBI), Miller Rocks Island (MRI), and East Sand Island (ESI). Bird species include Caspian terns (CATE), double-crested cormorants (DCCO), and California and ring-billed gulls (LAXX). Error bars represent 95% credible intervals for total mortality and avian consumption.

Cumulative avian consumption probabilities for sub-yearling Chinook salmon smolts during out-migration from Lower Monumental Dam to Pacific Ocean were like, but slightly lower than, those for yearling Chinook salmon smolts, ranging annually from 0.04 (0.03–0.07) to 0.10 (0.07–0.15) during 2008–2017 (Fig 4). Small sample sizes of PIT-tagged sub-yearling Chinook salmon smolts in some years resulted in imprecise estimates of total smolt mortality and, consequently, comparisons of total mortality to mortality due to avian consumption were also relatively imprecise. As such, results should be interpreted cautiously and are only reported herein where estimates provide a modicum of certainty. During 2008–2012, relatively large numbers (> 20,000) (Table 1) of sub-yearling Chinook salmon smolts were PIT-tagged and comparisons of total mortality and mortality due to avian consumption in those years indicated that avian consumption accounted for the largest proportion of total mortality between Lower Monumental Dam and McNary Dam, with estimates ranging annually from 28% (11–59%) to 78% (32–98%; Fig 4), very similar to results for yearling Chinook salmon (Fig 3). Downstream of McNary Dam, avian consumption was a smaller component of total mortality, with avian consumption accounting for less than 20% of total mortality in most reaches and years during smolt out-migration to Bonneville Dam (Fig 4). Comparisons of total mortality and mortality due to avian consumption during passage from Lower Monumental Dam to Bonneville Dam during 2008–2012, years with larger samples sizes of available tagged fish, indicated avian consumption accounted for 12% (7–24%) to 23% (10–63%) of all mortality for sub-yearling Chinook salmon smolts (Fig 4). Results suggest that in most years avian consumption represented a relatively small proportion of total sub-yearling Chinook salmon smolt mortality. Again, estimates of total mortality from Bonneville Dam to the Pacific Ocean were not available, so the impacts of consumption by terns and cormorants nesting on East Sand Island relative to all sources of mortality for Chinook salmon smolts are unknown, but may have been more substantial given the greater susceptibility of sub-yearling Chinook salmon smolts to consumption by cormorants and terns in the estuary (Fig 4).

Colony-specific estimates of consumption of sub-yearling Chinook salmon smolts were very similar to those for yearling Chinook salmon smolts. Of the individual colonies and river reaches evaluated, consumption probabilities for sub-yearling Chinook salmon during passage from Lower Monumental Dam to McNary Dam were less than 0.03 for all colonies of birds in all years. Like yearling Chinook salmon, consumption probabilities by birds from individual colonies for sub-yearling Chinook salmon during passage from McNary Dam to John Day Dam and from John Day Dam to Bonneville Dam were less than 0.02 per colony, per year, probabilities that were amongst the lowest observed. Analogous to other salmonid populations, consumption of sub-yearling Chinook salmon during passage from John Day Dam to Bonneville Dam was the highest for gulls nesting on Miller Rocks Island but consumption probabilities were less than 0.03 in all years (Fig 4). Consumption by cormorants nesting on East Sand Island in the Columbia River estuary were the highest of the 14 individual colonies evaluated, with consumption probabilities at 0.04 of available sub-yearling Chinook salmon in several years (Fig 4). Consumption probabilities for Caspian terns nesting on East Sand Island, however, were like those for tern, cormorant, and gull colonies upstream of Bonneville Dam, at less than 0.02 in most years (Fig 4).

#### Sockeye salmon

**Fig 5:**
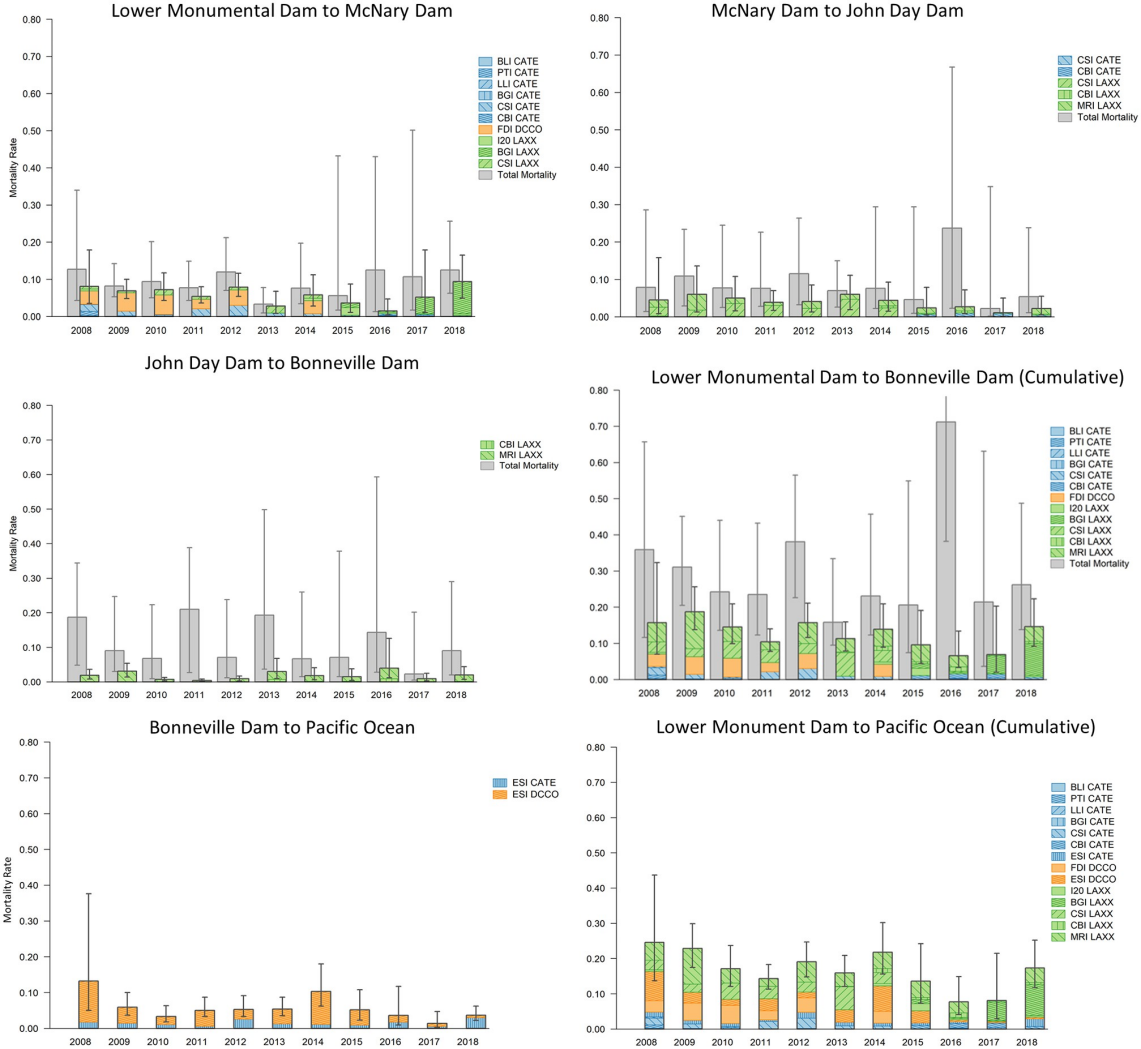
Avian consumption and total mortality of snake river sockeye salmon. Estimated annual colony-specific, reach-specific, and cumulative avian consumption probabilities (colored bars) and total mortality (grey bars) of Snake River sockeye salmon during passage from Lower Monumental Dam on the Snake River to the Pacific Ocean. Colony locations include Banks Lake Island (BLI), Potholes Reservoir (PTI), Lenore Lake Island (LLI), Island 20 (I20), Foundation Island (FDI), Badger Island (BGI), Crescent Island (CSI), Blalock Islands (BKI), Miller Rocks (MIR), and East Sand Island (ESI). Avian species include Caspian terns (CATE), double-crested cormorants (DCCO), and California and ring-billed gulls (LAXX). Error bars represent 95% credible intervals for total mortality and avian consumption.

Cumulative avian consumption probabilities for sockeye salmon smolts were consistently greater than those for yearling and sub-yearling Chinook salmon smolts, but often lower than those observed for steelhead smolts, with estimates ranging annually from 0.08 (0.03–0.22) to 0.25 (0.14–0.44) during passage from Lower Monumental Dam to the Pacific Ocean (Fig 5). Unlike avian consumption of steelhead, of the bird species evaluated, cumulative consumption probabilities for sockeye salmon smolts were often the highest by gull colonies, followed closely by cormorant colonies, with probabilities for gull colonies ranging annually from 0.05 (0.02–0.11) to 0.14 (0.08–0.22) and for cormorant colonies from 0.02 (0.01–0.04) to 0.10 (0.06–0.17; Fig 5). The cumulative effects of tern consumption on sockeye salmon smolts were generally low compared with those of gulls and cormorants, ranging annually from 0.02 (0.01–0.03) to 0.05 (0.02–0.10; Fig 5). Analogous to results for sub-yearling Chinook salmon, small sample sizes of PIT-tagged sockeye salmon smolts resulted in imprecise estimates of total mortality and, consequently, relative comparisons of total mortality to avian consumption. In the case of sockeye salmon, the lower 95% CRI bounds associated with estimates of total mortality exceeded the upper 95% CRI bound associated with estimates of cumulative avian consumption in several river reaches, and years evaluated. Due to such high levels of uncertainty, results should be interpreted cautiously and only general statements regarding relative comparisons of total mortality and mortality due to avian consumption on sockeye salmon smolts are provided herein. With this caveat in mind, comparisons of total mortality and mortality due to avian consumption suggest consumption effects were the greatest between Lower Monumental Dam and McNary Dam, with avian consumption accounting for roughly 10% to 80% of all sources of sockeye salmon smolt mortality annually (Fig 5). Estimates were often lower during smolt passage from McNary Dam to John Day Dam and from John Day Dam to Bonneville Dam, although point estimates of consumption by gulls nesting on Miller Rocks resulted in estimates of avian consumption accounting for more than 50% of all sockeye salmon smolt mortaality in some years (Fig 5). Estimates of cumulative total mortality for sockeye salmon smolts from Lower Monumental Dam to Bonneville Dam ranged annually from 0.16 (0.10–0.33) to 0.71 (0.38–0.88; Fig 5). Gross comparisons of total mortality to avian consumption indicated that avian consumption accounted for more than 50% of all sockeye salmon mortality during passage from Lower Monumental Dam to Bonneville Dam in 5 of the 11 years evaluated. Results suggest that the cumulative effects of avian consumption were generally greater for sockeye salmon than for yearling and sub-yearling Chinook salmon (i.e., the other salmon species evaluated). An appreciable proportion of sockeye salmon smolts (ca. 0.03 to 0.11 during 2008–2015) were depredated by cormorants breeding on East Sand Island in the Columbia River estuary (Fig 5), but analogous to the other salmonid populations evaluated, comparisons with total mortality were not available in this river reach.

Of the individual colonies evaluated, consumption probabilities for sockeye salmon smolts during passage from Lower Monumental Dam to McNary Dam were consistently the highest for cormorants nesting on Foundation Island. Consumption probabilities for Foundation Island cormorants preying on sockeye salmon smolts were consistently greater than 0.03, and as high as 0.05 (0.03–0.08; Fig 5). Consumption by gulls nesting on Badger Island and at other nearby gull colonies (i.e., Crescent Island and Island 20) were less than 0.02 in most years, except in 2017–2018, when upwards of 0.09 (0.04–0.16) of available sockeye salmon smolts were consumed by gulls nesting at the colony on Badger Island (Fig 5), shortly after the colony became established in 2015 (see also [7]). Consumption by terns nesting on Crescent Island were as high 0.03 during 2008–2014, prior to management to eliminate the colony in 2015. Consumption probabilities on sockeye salmon smolts during passage from McNary Dam to John Day Dam and from John Day Dam to Bonneville Dam were almost exclusively by gulls nesting on the Blalock Islands and on Miller Rocks. Unlike the consumption on steelhead, however, consumption by Central Blalock Islands terns on sockeye salmon smolts was low (less than 0.01; Fig 5), even during 2015–2018, when upwards of 0.08 of steelhead were consumed by terns nesting on the at Central Blalock Islands (Fig 2). Similar to consumption on both yearling and sub-yearling Chinook salmon, consumption by cormorants nesting on East Sand Island in the Columbia River estuary on sockeye salmon was amongst the highest of the 14 individual colonies evaluated, with estimates as high as 0.09 (0.05–0.17) in 2014 and 0.11 (0.04–0.34) in 2008 (Fig 5), estimates that were documented prior to mass colony dispersal events during the 2016–2018 nesting seasons (see Methods). Small sample sizes of PIT-tagged sockeye salmon smolts resulted in imprecise estimates of consumption probabilities in this river reach relative to the other salmonid populations evaluated, as indicated by the size of the 95% credible intervals (Fig 5).

## Discussion

The proportion of total smolt mortality during out-migration that was related to avian consumption was highly variable across salmonid species. Quantifying species-specific mortality factors is crucial to the development of species-specific recovery plans for ESA-listed salmonid populations in the Columbia River basin [2]. However, in multi predator-prey species systems it is important to identify how consumption varies across prey populations to accurately predict community-level responses. Our approach to jointly investigate multiple bird and prey species that share a common migration corridor revealed several important generalities, including (1) avian consumption was associated with the majority of mortality for steelhead smolts during out-migration, but a relatively minor proportion of total mortality for yearling and subyearling Chinook salmon smolts; and (2) the species, colony location, and colony size of piscivorous waterbirds nesting in the Columbia River basin dramatically influenced the magnitude of consumption, with some colonies posing little threat to smolt survival, while others were associated with mortality of a large proportion of the available fish.

Avian consumption was associated with a substantial proportion of all mortality for ESA-listed Snake River steelhead during the smolt life-stage, with the cumulative effects of avian consumption consistently greater than 0.20 (or 20%), and as high as 0.52 (or 52%), during 2008–2018. The annual cumulative probabilities of avian consumption of ESA-listed Upper Columbia River (UCR) steelhead smolts reported by Evans et al. [7] were very similar to those reported herein for Snake River steelhead smolts, with annual UCR steelhead consumption probabilities ranging between 0.31 and 0.53 of available smolts during 2008–2018. Like Snake River steelhead, UCR steelhead pass through the foraging range of multiple piscivorous waterbird colonies during seaward migration. Collectively, results suggest that avian consumption, particularly consumption by Caspian terns, was the single greatest direct source of steelhead mortality during out-migration, with avian consumption associated with more than 50% of all smolt mortality in 9 of the 11 years evaluated. Results of this and several other studies [4, 5, 7, 8, 15, 25], suggest that avian consumption, although not the original cause of salmonid declines in the Columbia River basin [32], is now a substantial mortality factor for ESA-listed steelhead smolts in the Columbia River basin.

The cumulative effects of avian consumption for Snake River sockeye salmon and Chinook salmon smolts were significantly lower than those for Snake River steelhead smolts in most river reaches, and years evaluated. Several other studies have documented that steelhead smolts are relatively more susceptible to consumption by colonial waterbirds than salmon smolts [4, 6, 8, 25, 33]. Potential reasons for the greater susceptibility of juvenile steelhead to avian consumption include differences in the size and behavior of steelhead smolts compared with smolts of other salmonid species [6, 25]. Steelhead smolts are on average larger (fork-length) and tend to be more surface-oriented compared with salmon smolts [3, 34], traits that make steelhead especially susceptible to plunge-diving and surface-snatching foragers like terns and gulls [20, 22–23].

Mortality associated with avian consumption compared with total smolt mortality for Chinook salmon smolts indicated that avian consumption was associated with a small proportion of all smolt mortality during out-migration to Bonneville Dam. These results are consistent with those of Evans et al. [25] and indicate that factors other than avian consumption were responsible for most of the mortality for Snake River yearling and sub-yearling Chinook salmon smolts during passage through the impounded section of the Snake and Columbia rivers. One component of Chinook salmon smolt mortality upstream of Bonneville Dam was likely consumption by piscivorous fishes, such as northern pikeminnow (*Ptychocheilus oregonensis*), smallmouth bass (*Micropterus dolomieu*), walleye (*Sander vitreus*), and channel catfish (*Ictalurus punctatus*). Rieman et al. [35] estimated that approximately 0.14 of juvenile salmonids passing through John Day Reservoir were consumed by fish and that mortality rates were highest for Chinook salmon relative to other salmonid species. Harnish et al. [36] and McMichael et al. [37] reported increases in the abundance of piscine predators in the Columbia River upstream of Bonneville Dam and hypothesized that piscine predation was the greatest direct source of sub-yearling Chinook salmon smolt mortality. Estimates of Chinook salmon smolt mortality associated with the direct effects of dam passage vary by age-class, dam, and year, with estimates ranging annually from approximately 0.01 to 0.06 of available Chinook salmon smolts per dam or 0.08 to 0.48 for those smolts that most pass all eight dams on the lower Snake and Columbia rivers combined [38–41]. In addition to piscine predators and direct mortality associated with dam passage, other direct sources of mortality for Chinook salmon smolts include mortality associated with disease, poor water quality, and other factors, but data to quantify these other sources of mortality are generally lacking [13].

Exceptions to the low probabilities of consumption by colonial waterbirds of Chinook salmon smolts were consumption probabilities for gulls nesting at colonies on Crescent Island and Miller Rocks Island and, especially, consumption probabilities by cormorants nesting on East Sand Island in the Columbia River estuary. Potential reasons for higher consumption probabilities for Chinook salmon by gulls and cormorants from these particular colonies may be related to the large size of these colonies (thousands of breeding pairs; [7, 42], behavioral flexibility to exploit temporarily available food sources [10, 21–23], or, in the case of gulls, the close proximity of breeding colonies to dams where smolts may be more vulnerable to consumption due to delays in passage, injury, mortality associated with turbine passage, or smolts being temporarily stunned or disoriented by hydraulic conditions in the tailrace of dams [25, 43, 44]. Gulls are also known to consume dead fish and to kleptoparasitize (steal) fish from other waterbirds, like terns, so probabilities for smolt consumption by gulls may not be indicative of predation probabilities [22, 25]. Unlike gulls, however, cormorants are strictly piscivorous and are not known to consume dead fish [21], so Chinook salmon smolt losses to cormorants nesting in the estuary, where probabilities were consistently the highest of the 14 colonies evaluated, may be especially concerning as predation occurs on individuals that have survived freshwater out-migration and are significantly more likely to return as adults compared to those yet to complete out-migration to the estuary [45].

Few studies have documented and quantified cause-specific mortality rates for Snake River sockeye salmon smolts during out-migration [46], making the results from this study novel regarding this critically endangered salmonid population [2]. Cumulative effects of avian consumption of Snake River sockeye salmon were substantial and represented a large proportion of all mortality of sockeye salmon smolts upstream of Bonneville Dam in several, but not all, years. This finding was unexpected, as previous research indicated that, relative to other juvenile salmonid species, sockeye salmon smolts comprised a small proportion of the diet of piscivorous colonial waterbirds in the Columbia River basin [10, 45, 47]. Previously published estimates of avian consumption have indicated similar levels of consumption on sockeye salmon and Chinook salmon smolts by cormorants and terns nesting at colonies in the basin, but datasets were limited to just one or two migration years and there were no estimates of the cumulative effects of birds from multiple breeding colonies, including gull colonies [4, 6, 8, 25]. Although small sample sizes of PIT-tagged sockeye salmon smolts resulted in imprecise estimates of both consumption and survival probabilities, sample sizes were sufficient to conclude that the cumulative effects of avian consumption on Snake River sockeye salmon smolts were consistently greater than the cumulative effects of avian consumption on Snake River yearling and sub-yearling Chinook salmon smolts in most river reaches and years evaluated. On average, sockeye salmon smolts are larger (fork-length) than sub-yearling Chinook salmon smolts, but generally smaller than yearling Chinook salmon smolts [3], so factors other than fish size may be related to the greater susceptibility to avian consumption of sockeye salmon smolts compared to Chinook salmon smolts. For example, differences in the run-timing of Snake River sockeye salmon smolts compared to Snake River yearling and sub-yearling Chinook salmon smolts may increase the susceptibility of sockeye salmon to avian consumption, with sockeye salmon smolt abundance in-river peaking after most yearling Chinook salmon smolts have out-migrated, but before most sub-yearling Chinook salmon smolts have out-migrated [48]. Peak abundance of sockeye salmon smolts in the lower Snake River and lower Columbia River also generally occurs during late-May, which coincides with the peak in colony attendance by piscivorous colonial waterbirds in the Columbia River basin [42].

Estimates of avian consumption probabilities presented herein represent minimum estimates of the total smolt mortality associated with piscivorous waterbirds in the Columbia River basin. Several key factors leading to minimum estimates include (1) not all piscivorous waterbird species in the basin were monitored, (2) not all bird colonies included in the study were searched for smolt PIT tags in all years, and (3) some bird colonies experienced abandonment within a nesting season, preventing on-colony PIT tag recoveries from representing total consumption by birds associated with the colony. For example, estimated smolt consumption probabilities for cormorants nesting at the colony on East Sand Island in the Columbia River estuary were biased low to an unknown degree due to colony abandonment events during 2016–2018, whereby cormorants temporarily dispersed from their nest sites, but remained in the Columbia River estuary in large numbers (several thousand individuals) during the peak of the smolt out-migration period [7]. Other breeding colonies of double-crested cormorants also exist on bridges and channel markers in the estuary, including a large colony (several thousand pairs) on the Astoria-Megler Bridge [7], a colony site located 13 Rkm upstream from East Sand Island where smolt PIT tags could not be recovered following the breeding season.

Several other piscivorous waterbird species also forage on juvenile salmonids in the Columbia River basin, species that were not included in this study. For example, we did not investigate impacts of consumption on smolts by American white pelicans (*Pelecanus erythrorthynchos)*, brown pelicans (*P*. *occidentalis*), Brandt’s cormorants (*Urile penicillatus*), glaucous-winged/western gulls (*L*. *glaucescens X L*. *occidentalis*), common mergansers (*Mergus merganser*), Forster’s terns (*Sterna forsteri*), great blue herons (*Ardea herodias*), black-crowned night-herons (*Nycticorax nycticorax*), and western and Clark’s grebes (*Aechmophorus* spp.). In the case of American white pelicans, minimum estimates of smolt consumption have been documented in other studies and suggest that impacts on steelhead and yearling Chinook salmon smolts were generally low (less than 0.01; [6]), but consumption probabilities for sub-yearling Chinook salmon smolts from certain stocks were significantly higher at upwards of 0.10 [49]; consumption probabilities for Snake River sockeye salmon smolts by pelicans are currently unknown. In the case of Brandt’s cormorants, which nest in the Columbia River estuary, consumption probabilities on smolts were consistently less than 0.01 of available smolts [24]. Unlike double-crested cormorants, Brandt’s cormorant primarily forage in marine waters where non-salmonid prey types are more common as compared to the species composition in estuary or river environments [50]. Consumption probabilities for glaucous-winged/western gulls, which also nest at colonies in the Columbia River estuary, are currently unknown but may be relatively small based on smaller colony sizes and the low proportion of salmonids reported in the diet of gulls nesting in the estuary (ca. 4%; [10]). In the case of non-colonial or semi-colonial piscivorous waterbirds (i.e., Forster’s terns, mergansers, herons, and grebes) documented consumption rates on salmonid smolts were much less than those of colonial piscivorous waterbirds [51], suggesting that these bird species pose a much smaller risk to smolt survival compared with colonies of Caspian terns, double-crested cormorants, and California and ring-billed gulls.

In addition to biotic factors like bird species, bird colony locations, bird abundance, fish size, and fish condition [52, 53], abiotic factors can also influence the susceptibility of juvenile salmonids to bird consumption. Petrosky and Schaller [54] observed a relationship between increased river flows and higher rates of steelhead smolt survival, a relationship that has been linked to variable consumption rates by colonial waterbirds, whereby higher river flows decrease fish travel times and consequently lower the exposure of smolts to bird consumption. For example, Hostetter et al. [52] observed that increased river flows were related to a decrease in Caspian tern consumption rates on steelhead smolts originating from the Snake River. Payton et al. [55] observed that shorter water transit times (a measure of flow in relation to reservoir levels) were associated with lower consumption rates by Caspian terns on steelhead smolts passing through the Wanapum and Priest Rapids reservoirs in the middle Columbia River. Collectively, results from these and other studies indicate that both biotic factors and abiotic conditions experienced by juvenile salmonids during out-migration influence their susceptibility to avian consumption.

Our ability to investigate species-specific predator-prey interactions across 4 fish populations and 14 different bird colonies provides several important considerations for multi-predator multi-prey systems, including (1) highly variable predation among closely related prey populations, (2) the importance of predator spatial distributions, and (3) the need for systemwide studies to quantify multiple mortality sources. Many monitoring programs often focus on one or two surrogate or indicator species to track ecosystem processes across species with similar life histories [56]. Our findings provide a cautionary example of surrogate approaches, where for example monitoring of Chinook salmon populations may have overlooked high avian predation on steelhead populations, even though these species out-migrate through the same rivers during similar times of year. In most regions, scientists and managers lack detailed information on species-specific predator-prey relationships but nevertheless, must move forward with management decisions [57]. Intensively monitored and managed systems like the Columbia River hydrosystem are informative examples of resolving critical uncertainties regarding multi-species and ecosystem-level responses to management actions.

## Conclusion

Avian consumption was associated with a substantial proportion of total smolt mortality for Snake River steelhead. In contrast, cumulative avian consumption probabilities for Snake River yearling and sub-yearling Chinook salmon, were often low and represented a relatively small proportion of total mortality during smolt out-migration through the impounded sections of the Snake and Columbia rivers. The impact on Chinook salmon smolt survival from consumption by cormorants nesting at the large colony in Columbia River estuary was, however, similar to the cumulative risk to steelhead smolt survival from avian consumption. Our results suggest that the potential benefits of managing birds to reduce smolt mortality will vary greatly by the bird species (tern, cormorant, gull), by the breeding colony, and by the salmonid species and population. Collectively, these results and the analytical framework used to jointly estimate avian consumption related mortality and survival provide data to help prioritize where management actions directed at reducing smolt mortality from birds might be the most beneficial for recovery of ESA-listed salmonids and a method to quantify the effects of avian consumption on survival of salmonid smolts across large spatial-scales.

## Supporting information

S1 File Prior distribution parameter specifications used to identify probabilities of detecting depredated and deposited tags. Using data from other studies, the probability of detecting a deposited tag (p) for each colony in each year was modelled using logistic regression (logit(p) ~ β_1+β_2*(week-22)) and informed through the use of intentionally sown test tags. The resulting joint-posterior distribution of [β_1,β_2] ^T for each colony was approximated as a multivariate normal distribution, the values of which were employed as informative priors for use in this study. Those informative priors are provided here as supplemental materials and can be used to replicate study results.
(DOCX)
