# Supplementary material for: Cumulative effects of piscivorous colonial waterbirds on juvenile salmonids: A multi predator-prey species evaluation
Source: PLoS One. 2022 Aug 10;17(8):e0272875. doi: 10.1371/journal.pone.0272875 (PMC9365185; doi:10.1371/journal.pone.0272875)
Supplement: S1 File — Using data from other studies, the probability of detecting a deposited tag (p) for each colony in each year was modelled using logistic regression (logit(p) ~ β_1+β_2*(week-22)) and informed through the use of intentionally sown test tags. The resulting joint-posterior distribution of [β_1,β_2] ^T for each colony was approximated as a multivariate normal distribution, the values of which were employed as informative priors for use in this study. Those informative priors are provided here as supplemental materials and can be used to replicate study results. (DOCX) [file pone.0272875.s001.docx]

Using data from other studies, the probability of detecting a deposited tag (p) for each colony in each year was modelled using logistic regression (logit(p) ~ β_1+β_2*(week-22)) and informed through the use of intentionally sown test tags. The resulting joint-posterior distribution of [β_1,β_2 ]^T for each colony was approximated as a multivariate normal distribution, the values of which were employed as informative priors for use in this study. Those informative priors are provided here as supplemental materials and can be used to replicate study results.

A list raw tag codes sown on bird colonies to estimate detection probabilities or tags used in deposition studies are available upon request to the authors of those studies.

A map of Caspian tern (CATE), double-crested cormorant (DCCO), and California and ring-billed gull (LAXX) colony locations is provided in Figure 1. In some cases, multiple subcolonies existed on an island or chain of islands. Subcolonies are denoted 1, 2, 3.

| year | colony | mu1_1 | mu1_2 | cov1_1 | cov1_2 | cov2_1 | cov2_2 |
| --- | --- | --- | --- | --- | --- | --- | --- |
| 2008 | CATEBLI | 0.5026 | 0.439 | 0.7607 | 0.0301 | 0.0301 | 0.078 |
| 2008 | CATEPTI-1 | 0.5794 | 0.1231 | 0.1077 | 0.0016 | 0.0016 | 0.0195 |
| 2008 | DCCOFDI | 1.0452 | -0.0159 | 0.112 | 0.002 | 0.002 | 0.0163 |
| 2008 | CATECSI | 0.651 | 0.1721 | 0.0841 | 0.0025 | 0.0025 | 0.013 |
| 2008 | LAXXCSI | 1.6271 | 0.1503 | 0.2621 | 0.017 | 0.017 | 0.0198 |
| 2008 | CATECBI-1 | 3.3453 | 0.1463 | 0.7363 | 0.0604 | 0.0604 | 0.0455 |
| 2008 | LAXXMRI | 1.5883 | 0.0127 | 0.1986 | 0.0041 | 0.0041 | 0.0215 |
| 2008 | CATEESI | 2.5374 | 0.0671 | 0.1619 | 0.0033 | 0.0033 | 0.0207 |
| 2008 | DCCOESI | 0.6411 | 0.0612 | 0.1165 | -6.00E-04 | -6.00E-04 | 0.0099 |
| 2009 | CATEBLI | 1.6921 | 0.3376 | 0.6032 | 0.0685 | 0.0685 | 0.0468 |
| 2009 | CATEPTI-1 | -0.1549 | 0.0932 | 0.105 | -0.0014 | -0.0014 | 0.0178 |
| 2009 | DCCOFDI | 0.917 | -0.0345 | 0.1179 | 0.0036 | 0.0036 | 0.0155 |
| 2009 | CATECSI | 1.4398 | 0.2639 | 0.1665 | 0.0146 | 0.0146 | 0.0233 |
| 2009 | LAXXCSI | 1.0843 | 0.0758 | 0.179 | 0.0057 | 0.0057 | 0.0189 |
| 2009 | CATECBI-1 | 2.9108 | 0.214 | 0.7267 | 0.0653 | 0.0653 | 0.0345 |
| 2009 | LAXXMRI | 1.4275 | 0.0641 | 0.2035 | 0.0079 | 0.0079 | 0.0198 |
| 2009 | CATEESI | 2.3165 | 0.0491 | 0.1476 | 0 | 0 | 0.0161 |
| 2009 | DCCOESI | 0.6468 | 0.0145 | 0.089 | -0.002 | -0.002 | 0.007 |
| 2010 | CATEBLI | 1.7226 | 0.3353 | 0.6808 | 0.0606 | 0.0606 | 0.0425 |
| 2010 | CATEPTI-1 | 0.208 | 0.5061 | 0.1417 | -0.0023 | -0.0023 | 0.0651 |
| 2010 | DCCOFDI | 0.5536 | 0.0086 | 0.1095 | 0.0034 | 0.0034 | 0.0169 |
| 2010 | CATECSI | 1.5426 | 0.2478 | 0.1617 | 0.0125 | 0.0125 | 0.0223 |
| 2010 | LAXXCSI | 1.4396 | 0.077 | 0.2039 | 0.0081 | 0.0081 | 0.0215 |
| 2010 | CATECBI-1 | 1.2961 | 0.0661 | 0.7702 | -0.0745 | -0.0745 | 0.0523 |
| 2010 | LAXXMRI | 1.4338 | 0.1134 | 0.2118 | 0.0129 | 0.0129 | 0.0222 |
| 2010 | CATEESI | 1.572 | 0.0786 | 0.1384 | 0.0015 | 0.0015 | 0.0199 |
| 2010 | DCCOESI | 1.0739 | 0.0478 | 0.1203 | -1.00E-04 | -1.00E-04 | 0.0111 |
| 2011 | CATEPTI-1 | 0.3288 | 0.2528 | 0.1285 | 9.00E-04 | 9.00E-04 | 0.0248 |
| 2011 | CATEBGI | 1.1107 | 0.0342 | 0.5216 | -0.0202 | -0.0202 | 0.0191 |
| 2011 | DCCOFDI | -0.0366 | 0.0583 | 0.158 | -0.0022 | -0.0022 | 0.0114 |
| 2011 | CATECSI | 1.6548 | 0.1529 | 0.2206 | 0.0157 | 0.0157 | 0.0261 |
| 2011 | LAXXCSI | 0.982 | 0.0972 | 0.2573 | 0.0099 | 0.0099 | 0.0253 |
| 2011 | CATECBI-1 | 1.2961 | 0.0661 | 0.7702 | -0.0745 | -0.0745 | 0.0523 |
| 2011 | LAXXMRI | 1.4524 | 0.0551 | 0.2846 | 0.0105 | 0.0105 | 0.0311 |
| 2011 | CATEESI | 1.2127 | 0.0423 | 0.1376 | 0.0011 | 0.0011 | 0.0189 |
| 2011 | DCCOESI | 0.9378 | 0.001 | 0.1218 | -0.0035 | -0.0035 | 0.0089 |
| 2012 | CATEBLI | 1.1483 | 0.1142 | 0.511 | -0.0711 | -0.0711 | 0.0733 |
| 2012 | CATEPTI-1 | -0.0763 | 0.2095 | 0.1221 | -0.0018 | -0.0018 | 0.0213 |
| 2012 | CATEBGI | 0.8709 | 0.0253 | 0.2554 | 0.0087 | 0.0087 | 0.0236 |
| 2012 | DCCOFDI | -0.5157 | 0.0134 | 0.1513 | -0.0032 | -0.0032 | 0.0113 |
| 2012 | CATECSI | 0.9261 | 0.1885 | 0.1825 | 0.0103 | 0.0103 | 0.0276 |
| 2012 | LAXXCSI | 1.581 | 0.2035 | 0.4218 | 0.0363 | 0.0363 | 0.0335 |
| 2012 | LAXXMRI | 1.6115 | 0.0938 | 0.3076 | 0.0147 | 0.0147 | 0.0297 |
| 2012 | CATEESI | 1.094 | 0.1004 | 0.2194 | 0.0041 | 0.0041 | 0.0184 |
| 2012 | DCCOESI | 0.8622 | 0.047 | 0.167 | -0.0019 | -0.0019 | 0.0137 |
| 2013 | CATEPTI-1 | 0.4793 | 0.1991 | 0.1214 | 0.0015 | 0.0015 | 0.0203 |
| 2013 | LAXXIS20 | 1.4733 | 0.0988 | 0.2354 | 0.0154 | 0.0154 | 0.0271 |
| 2013 | CATECSI | 1.5819 | 0.1726 | 0.2272 | 0.014 | 0.014 | 0.0282 |
| 2013 | LAXXCSI | 1.1373 | 0.1199 | 0.2187 | 0.013 | 0.013 | 0.0275 |
| 2013 | CATECBI-1 | 1.2872 | 0.055 | 0.9069 | -0.0784 | -0.0784 | 0.0461 |
| 2013 | LAXXMRI | 1.6475 | 0.0701 | 0.2469 | 0.0136 | 0.0136 | 0.0287 |
| 2013 | LAXXCBI-1 | 1.591 | 0.0706 | 0.2385 | 0.0115 | 0.0115 | 0.0295 |
| 2013 | LAXXCBI-2 | 0.626 | 0.1956 | 0.2291 | 0.0153 | 0.0153 | 0.03 |
| 2013 | CATEESI | 0.2805 | 0.0503 | 0.1411 | -0.0047 | -0.0047 | 0.0109 |
| 2013 | DCCOESI | 0.7253 | 0.016 | 0.1193 | -0.0028 | -0.0028 | 0.0079 |
| 2014 | CATEBLI | 1.1483 | 0.1142 | 0.511 | -0.0711 | -0.0711 | 0.0733 |
| 2014 | CATEPTI-1 | 0.317 | 0.3509 | 0.4806 | -0.0291 | -0.0291 | 0.0903 |
| 2014 | LAXXIS20 | 1.6417 | 0.0897 | 0.489 | -0.0246 | -0.0246 | 0.0446 |
| 2014 | DCCOFDI | -1.393 | 0 | 0.2575 | 0 | 0 | 0 |
| 2014 | CATECSI | 1.8192 | 0.1242 | 0.2257 | 0.0131 | 0.0131 | 0.0299 |
| 2014 | LAXXCSI | 2.2875 | 0.2555 | 0.5373 | 0.0662 | 0.0662 | 0.0562 |
| 2014 | CATECBI-1 | 1.2872 | 0.055 | 0.9069 | -0.0784 | -0.0784 | 0.0461 |
| 2014 | LAXXMRI | 1.7218 | 0.0259 | 0.2939 | 0.0045 | 0.0045 | 0.0452 |
| 2014 | LAXXCBI-1 | 2.9732 | 0.1557 | 0.5813 | 0.0575 | 0.0575 | 0.0729 |
| 2014 | LAXXCBI-2 | 2.7954 | 0.1802 | 0.5978 | 0.0636 | 0.0636 | 0.0638 |
| 2014 | CATEESI | 0.565 | 0.0746 | 0.1305 | 0.0011 | 0.0011 | 0.015 |
| 2014 | DCCOESI | 1.0993 | 0.0426 | 0.1184 | -5.00E-04 | -5.00E-04 | 0.0104 |
| 2015 | CATEBLI | 1.1682 | 0.093 | 0.2693 | 0.0164 | 0.0164 | 0.028 |
| 2015 | LAXXIS20 | 1.7618 | 0.0532 | 0.293 | 0.0146 | 0.0146 | 0.0351 |
| 2015 | LAXXBGI | 1.3674 | 0.0709 | 0.572 | -0.0196 | -0.0196 | 0.0282 |
| 2015 | CATECBI-1 | 1.2862 | 0.1224 | 0.4268 | 0.0294 | 0.0294 | 0.0399 |
| 2015 | CATECBI-2 | 1.4746 | 0.1578 | 0.4812 | 0.0366 | 0.0366 | 0.0426 |
| 2015 | CATECBI-3 | 1.2755 | 0.1193 | 0.4166 | 0.0287 | 0.0287 | 0.0409 |
| 2015 | LAXXMRI | 2.1708 | 0.1196 | 0.4175 | 0.0282 | 0.0282 | 0.031 |
| 2015 | LAXXCBI-1 | 3.06 | 0.1368 | 0.6343 | 0.0509 | 0.0509 | 0.0404 |
| 2015 | LAXXCBI-2 | 2.5933 | 0.1825 | 0.5776 | 0.053 | 0.053 | 0.0401 |
| 2015 | CATEESI | 1.6656 | 0.0871 | 0.1841 | -0.0011 | -0.0011 | 0.0192 |
| 2015 | DCCOESI | 1.2895 | 0.0383 | 0.1263 | -0.0013 | -0.0013 | 0.0101 |
| 2016 | CATEBLI | 1.3962 | 0.1448 | 0.3054 | 0.0181 | 0.0181 | 0.0327 |
| 2016 | CATEPTI-1 | 1.105 | 0.0461 | 0.3533 | 0.0236 | 0.0236 | 0.1285 |
| 2016 | CATEPTI-2 | 1.105 | 0.0461 | 0.3533 | 0.0236 | 0.0236 | 0.1285 |
| 2016 | CATELLI | 1.2052 | 0.0242 | 0.6198 | -0.0374 | -0.0374 | 0.0382 |
| 2016 | LAXXIS20 | 1.3178 | 0.0395 | 0.2642 | 0.0093 | 0.0093 | 0.031 |
| 2016 | LAXXBGI | 0.4018 | 0.0043 | 0.6609 | -0.0302 | -0.0302 | 0.0159 |
| 2016 | CATECBI-1 | 1.0841 | 0.0957 | 0.2573 | 0.0082 | 0.0082 | 0.0312 |
| 2016 | CATECBI-2 | 1.0562 | 0.1489 | 0.2748 | 0.0131 | 0.0131 | 0.034 |
| 2016 | CATECBI-3 | 1.0923 | 0.0972 | 0.244 | 0.0067 | 0.0067 | 0.0315 |
| 2016 | LAXXMRI | 1.5731 | 0.0147 | 0.262 | 0.0028 | 0.0028 | 0.0267 |
| 2016 | LAXXCBI-1 | 2.1629 | -0.0111 | 0.3419 | -6.00E-04 | -6.00E-04 | 0.0398 |
| 2016 | LAXXCBI-2 | 2.9175 | 0.179 | 0.6226 | 0.0567 | 0.0567 | 0.0432 |
| 2016 | CATEESI | 1.1833 | 0.0778 | 0.1592 | -0.0024 | -0.0024 | 0.0147 |
| 2016 | DCCOESI | 0.3999 | 0.0033 | 0.1267 | -0.0032 | -0.0032 | 0.0098 |
| 2017 | CATELLI | 1.2332 | 0.2038 | 0.5142 | -0.0309 | -0.0309 | 0.0724 |
| 2017 | LAXXIS20 | 1.6363 | 0.03 | 0.2884 | 0.0102 | 0.0102 | 0.0277 |
| 2017 | CATEBGI | 2.4901 | 0.0616 | 0.9662 | -0.0147 | -0.0147 | 0.0384 |
| 2017 | LAXXBGI | 1.2058 | 0.1088 | 0.3621 | 0.0158 | 0.0158 | 0.0188 |
| 2017 | CATECBI-1 | 0.3584 | 0.0448 | 0.2227 | -0.0032 | -0.0032 | 0.0178 |
| 2017 | CATECBI-2 | -0.2317 | 0.0679 | 0.2155 | -0.0062 | -0.0062 | 0.0177 |
| 2017 | CATECBI-3 | 0.3558 | 0.0444 | 0.2099 | -0.0031 | -0.0031 | 0.0169 |
| 2017 | LAXXMRI | 1.1454 | 0.0724 | 0.2512 | 0.0066 | 0.0066 | 0.0278 |
| 2017 | LAXXCBI-1 | 3.1255 | 0.1423 | 0.6473 | 0.0552 | 0.0552 | 0.0498 |
| 2017 | LAXXCBI-2 | 2.4254 | 0.0158 | 0.3532 | 0.0051 | 0.0051 | 0.0402 |
| 2017 | CATEESI | 0.8512 | 0.0957 | 0.1435 | -8.00E-04 | -8.00E-04 | 0.0177 |
| 2017 | DCCOESI | 0.8306 | 0.0364 | 0.1143 | 0 | 0 | 0.0092 |
| 2018 | CATELLI | -0.5782 | 0.3235 | 0.9304 | -0.0036 | -0.0036 | 0.0676 |
| 2018 | LAXXIS20 | 3.2603 | 0.1204 | 0.6913 | 0.0511 | 0.0511 | 0.0469 |
| 2018 | LAXXBGI | -0.6137 | 0.0196 | 0.2243 | -0.0029 | -0.0029 | 0.0126 |
| 2018 | CATECBI-1 | -0.615 | 0.0689 | 0.2383 | -0.0071 | -0.0071 | 0.0172 |
| 2018 | CATECBI-2 | -0.2707 | 0.0628 | 0.2264 | -0.0049 | -0.0049 | 0.0177 |
| 2018 | CATECBI-3 | -0.6301 | 0.0698 | 0.2427 | -0.0075 | -0.0075 | 0.0177 |
| 2018 | LAXXMRI | 1.8845 | 0.1017 | 0.3482 | 0.0188 | 0.0188 | 0.0355 |
| 2018 | LAXXCBI-1 | 3.1641 | 0.164 | 0.662 | 0.0628 | 0.0628 | 0.0573 |
| 2018 | LAXXCBI-2 | 2.1499 | 0.1064 | 0.373 | 0.0252 | 0.0252 | 0.0421 |
| 2018 | CATEESI | 1.1628 | 0.1431 | 0.2182 | 0.0127 | 0.0127 | 0.0238 |
| 2018 | DCCOESI | 2.3827 | 0.1291 | 0.3946 | 0.0309 | 0.0309 | 0.0276 |
